# TMEM2 is a *bona fide* hyaluronidase possessing intrinsic catalytic activity

**DOI:** 10.1016/j.jbc.2023.105120

**Published:** 2023-07-30

**Authors:** Takuma Narita, Yuki Tobisawa, Andrey Bobkov, Michael Jackson, Chikara Ohyama, Fumitoshi Irie, Yu Yamaguchi

**Affiliations:** 1 Human Genetics Program, Sanford Burnham Prebys Medical Discovery Institute, La Jolla, California, USA; 2 Department of Urology, Hirosaki University Graduate School of Medicine, Hirosaki, Japan; 3 Conrad Prebys Center for Chemical Genomics, Sanford Burnham Prebys Medical Discovery Institute, La Jolla, California, USA

**Keywords:** TMEM2, hyaluronan, hyaluronidase, enzyme, enzyme purification, recombinant protein expression, HYAL2

## Abstract

Transmembrane protein 2 (TMEM2) was originally identified as a membrane-anchored protein of unknown function. We previously demonstrated that TMEM2 can degrade hyaluronan (HA). Furthermore, we showed that induced global knockout of *Tmem2* in adult mice results in rapid accumulation of incompletely degraded HA in bodily fluids and organs, supporting the identity of TMEM2 as a cell surface hyaluronidase. In spite of these advances, no direct evidence has been presented to demonstrate the intrinsic hyaluronidase activity of TMEM2. Here, we directly establish the catalytic activity of TMEM2. The ectodomain of TMEM2 (TMEM2^ECD^) was expressed as a His-tagged soluble protein and purified by affinity and size-exclusion chromatography. Both human and mouse TMEM2^ECD^ robustly degrade fluorescein-labeled HA into 5 to 10 kDa fragments. TMEM2^ECD^ exhibits this HA-degrading activity irrespective of the species of TMEM2 origin and the position of epitope tag insertion. The HA-degrading activity of TMEM2^ECD^ is more potent than that of HYAL2, a hyaluronidase which, like TMEM2, has been implicated in cell surface HA degradation. Finally, we show that TMEM2^ECD^ can degrade not only fluorescein-labeled HA but also native high-molecular weight HA. In addition to these core findings, our study reveals hitherto unrecognized confounding factors, such as the quality of reagents and the choice of assay systems, that could lead to erroneous conclusions regarding the catalytic activity of TMEM2. In conclusion, our results demonstrate that TMEM2 is a legitimate functional hyaluronidase. Our findings also raise cautions regarding the choice of reagents and methods for performing degradation assays for hyaluronidases.

Hyaluronan (HA) is an extremely large polysaccharide of the glycosaminoglycan family, representing one of the major components of extracellular matrices. *Via* its unique biochemical and biophysical properties, HA plays key roles in regulating the size, fluid pressure, and malleability of tissues. HA and small HA fragments also exert a range of effects on cell behavior and signaling (1). *In vivo*, HA is continuously turned over with a short half-life. It is believed that bulky HA polymers in the extracellular space are first cleaved into intermediate-sized fragments, which are subsequently internalized and degraded into oligosaccharides and monosaccharides in endosomes and lysosomes (2). Although multiple proteins in different cellular compartments have been implicated in these catabolic processes, the identity of the hyaluronidase that acts on the cell surface has been uncertain. Although HYAL2 has been shown to be anchored to the cell surface *via* a glycosylphosphatidylinositol linkage (3, 4), its acidic pH optimum (5, 6) and the intracellular localization of endogenous HYAL2 protein (7) suggest that it may not be a full-time cell surface hyaluronidase. In addition, in spite of the rapid turnover of extracellular HA during embryonic development, *Hyal2*^*−/−*^ mice exhibit only mild embryonic phenotype (8, 9, 10).

In 2017, we reported that transmembrane protein 2 (TMEM2), a type II transmembrane protein of unknown function, is the sought-after cell surface hyaluronidase (11). Our subsequent studies have assembled multiple pieces of evidence supporting the physiological relevance of TMEM2 as a functional hyaluronidase. Specifically, we showed that: (i) tamoxifen-induced global knockout of *Tmem2* in adult mice leads to rapid and pronounced accumulation of uncleaved and partially cleaved HA in bodily fluids and a variety of organs (12); (ii) *Wnt1-Cre*–driven *Tmem2* conditional knockout results in severe defects in neural crest derivatives, accompanied by aberrant HA accumulation in those tissues (13); and (iii) knockdown of TMEM2 in human tumor cells greatly impairs their ability to degrade HA in a contact-dependent manner (14). Up until now, however, there has been no direct evidence demonstrating that the TMEM2 protein itself has intrinsic HA-degrading activity. The lack of such data appears to have caused some confusion and skepticism concerning whether TMEM2 is a functional hyaluronidase. In this paper, we present multiple pieces of evidence demonstrating that TMEM2 is a *bona fide* hyaluronidase that has intrinsic HA-degrading activity. This work also identifies technical factors that may confound the outcome of assays for TMEM2 enzyme activity.

## Results

### TMEM2 ectodomain has intrinsic hyaluronidase activity

**Figure 1:**
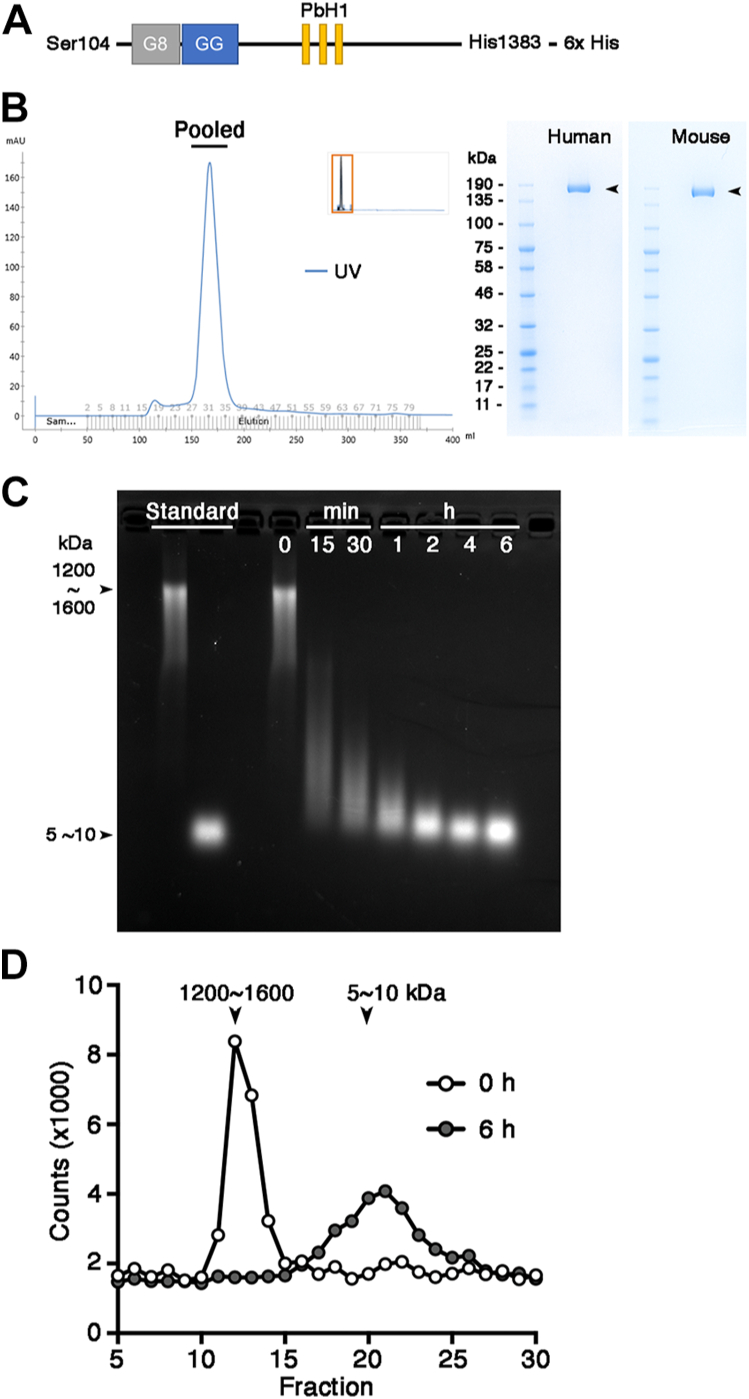
**Purified human TMEM2**^**ECD**^**possesses HA-degrading activity.***A*, structure of TMEM2^ECD^. *G8*, G8 domain; *GG*, GG domain; *PbH1*, PbH1 repeats. *B*, the final step of purification of human TMEM2^ECD^ by Superdex 200 size-exclusion chromatography. The peak fractions (*horizontal bar*) were pooled and analyzed by SDS-PAGE and Coomassie blue staining (*Human*). SDS-PAGE of mouse TMEM2^ECD^, isolated by the same procedure, is also shown (*Mouse*). *C* and *D*, HA degradation assay with human TMEM2^ECD^ analyzed by agarose gel electrophoresis (*C*) and size-exclusion chromatography on Sephacryl S-300HR (*D*). HA, hyaluronan; TMEM2, transmembrane protein 2; TMEM2^ECD^, ectodomain of TMEM2.

To determine whether TMEM2 has intrinsic hyaluronidase activity, we produced a recombinant soluble human TMEM2 protein representing its ectodomain (hereafter designated as TMEM2^ECD^). cDNA corresponding to Ser^104^–His^1383^ of human TMEM2, with a 6× His tag fused to its C terminus (Figs. 1*A* and S1), was transfected in FreeStyle 293-F cells, and TMEM2^ECD^ was purified from culture supernatants (Fig. 1*B*). HA degradation assays with purified human TMEM2^ECD^ were performed using fluorescein-labeled HA (hereafter designated as FA–HA) from Cosmo Bio (1200–1600 kDa) as a substrate, and the degradation of FA–HA was monitored by agarose gel electrophoresis. As shown in Figure 1*C*, FA–HA was completely degraded into 5 to 10 kDa fragments in 6 h, with the bulk of reaction occurring within 2 h. Analysis by size-exclusion chromatography yielded a similar result (Fig. 1*D*). These results directly demonstrate that the human TMEM2 ectodomain possesses intrinsic HA-degrading activity. Significantly, HA degradation by purified TMEM2^ECD^ occurs much more rapidly than the HA degradation observed in cell-based assays using TMEM2-transfected 293T cells (∼72 h; (11)), the system we originally employed to demonstrate the hyaluronidase activity of TMEM2. This large discrepancy in reaction rates suggests that the cell-based assays may be influenced by a component(s) that suppresses the activity of TMEM2 (see below for these data: Factors that may confound results obtained in TMEM2 hyaluronidase assays).

**Figure 2:**
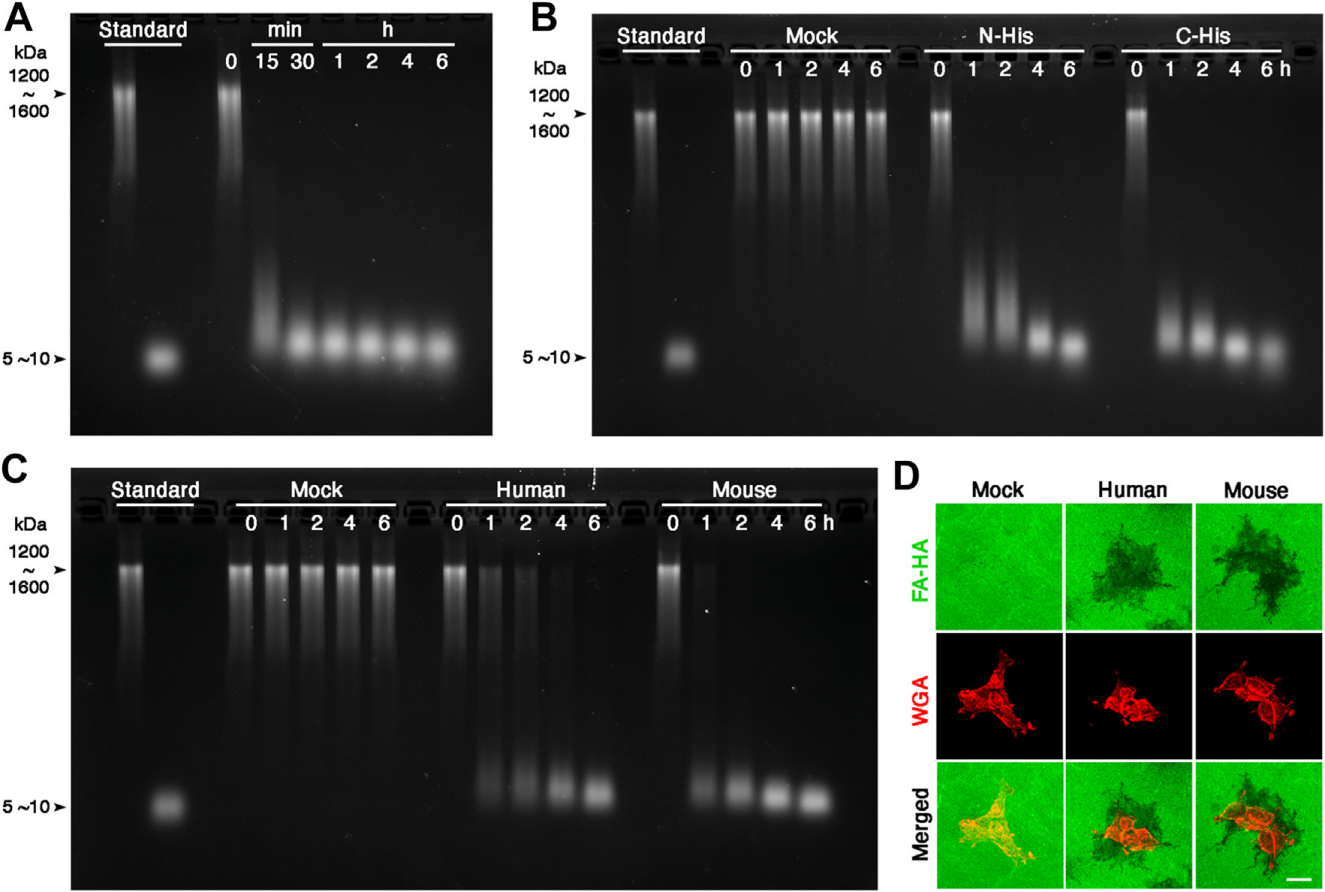
**Multiple recombinant TMEM2 species exhibit HA-degrading activity.***A*–*C*, HA degradation assays were performed with: (*A*) mouse TMEM2^ECD^; (*B*) N- and C-terminally tagged human TMEM2^ECD^; and (*C*) the P2 membrane fractions of 293T cells transfected with mouse and human full-length TMEM2. In *B*, concentrated culture supernatants of 293T cells transfected with respective constructs were used as enzyme sources. The amount of enzyme input was normalized by immunoblotting with anti-TMEM2 polyclonal antibody. Control reactions were performed with culture supernatants (*B*) and with the P2 fraction (*C*) of mock-transfected 293T cells (*Mock*). In *D*, the ability of mouse and human full-length TMEM2 to degrade substrate-bound FA–HA (*green*) was analyzed by *in situ* HA degradation assays with 293 cells transfected with mouse and human TMEM2. Cells were visualized by staining with Alexa Fluor 594-conjugated wheat germ agglutinin. Scale bar, 20 μm. FA–HA, fluorescein-labeled HA; HA, hyaluronan; TMEM2, transmembrane protein 2; TMEM2^ECD^, ectodomain of TMEM2.

To substantiate this initial finding, we performed four corroborating experiments using different recombinant TMEM2 proteins and assay systems. First, we purified mouse TMEM2^ECD^ with the same structural configuration as human TMEM2^ECD^ (Fig. 1*B*). As observed for human TMEM2^ECD^, mouse TMEM2^ECD^ exhibits robust HA-degrading activity (Fig. 2*A*). The time course of FA–HA degradation by mouse TMEM2^ECD^ is faster than that of human TMEM2^ECD^, the reaction being almost complete within 1 h. Second, we examined the possible effect of the position of the 6× His tag on the HA-degrading activity of TMEM2^ECD^. For this, we produced a second human TMEM2^ECD^ in which a 6× His tag was fused to its N terminus. This N-terminally tagged human TMEM2^ECD^ exhibits an HA-degrading activity that is nearly as robust as that of C-terminally fused TMEM2^ECD^ (Fig. 2*B*). Third, we examined whether full-length TMEM2 associated with cell membranes exhibits HA-degrading activity. For this, the HA degradation assays were performed with the isolated membrane fraction of TMEM2-transfected 293T cells as the enzyme source. These assays demonstrate that both human and mouse full-length TMEM2 degrade HA (Fig. 2*C*). As seen in the experiments with TMEM2^ECD^ (see Fig. 2*A*), mouse TMEM2 exhibits stronger activity than human TMEM2 in this assay system. Finally, the HA-degrading activities of both human and mouse full-length TMEM2 are further confirmed by *in situ* HA degradation assays using transfected 293 cells (Fig. 2*D*). Together, these results demonstrate that TMEM2 possesses intrinsic HA-degrading activity, independent of the species of TMEM2 origin, the position of epitope tag insertion, and whether or not it is anchored to cell membrane.

### The HA-degrading activity of TMEM2 is more potent than that of HYAL2

**Figure 3:**
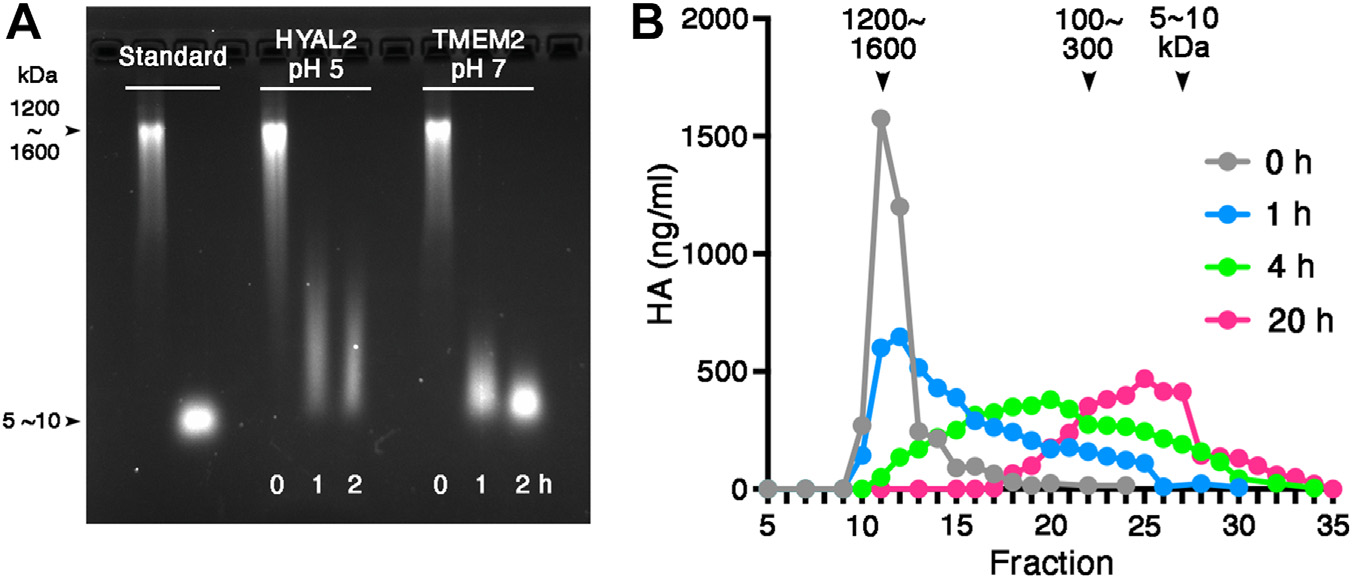
**TMEM2 possesses physiologically relevant HA-degrading activity.***A*, comparison of HA-degrading activity between HYAL2 and TMEM2^ECD^. Reactions were performed with an equimolar amount (33 nM) of recombinant human HYAL2 and human TMEM2^ECD^ proteins at the pH optima for respective enzymes. *B*, degradation of native, high-molecular weight HA with human TMEM2^ECD^. Degradation of HA was analyzed by size-exclusion chromatography on Sepharose CL-2B. HA, hyaluronan; TMEM2, transmembrane protein 2; TMEM2^ECD^, ectodomain of TMEM2.

To define the physiological importance of TMEM2 as a functional hyaluronidase, we performed two additional studies. First, we asked how the observed HA-degrading activity of TMEM2 compares with that of established hyaluronidases. To address this question, we chose to compare TMEM2 with HYAL2, which, like TMEM2, has been implicated in HA degradation on the cell surface (15, 16). The recombinant human HYAL2 (R&D Systems) used for this analysis is appropriate for the side-by-side comparison—like our human TMEM2^ECD^, it is produced as a soluble protein with a C-terminal 6× His tag. Assays were performed at the pH optima for respective enzymes (pH 5.0 for HYAL2 (17); pH 7.0 for TMEM2^ECD^ (11)). The results of the comparison demonstrate that while TMEM2^ECD^ degrades FA–HA completely into 5 to 10 kDa fragments in 2 h, the reaction by HYAL2 does not appear to reach completion at the same time point (Fig. 3*A*). Moreover, the degradation products of HYAL2 action are much larger (ranging from 100 to 300 kDa) and more polydisperse than those produced by TMEM2^ECD^. These results indicate that the rate of cleavage is faster for TMEM2^ECD^ than for HYAL2 and that TMEM2^ECD^ cleaves HA polymers at many more sites than HYAL2. Assuming, for example, that the average molecular size of the fragments generated by TMEM2^ECD^ is 10 kDa and that of HYAL2 is 200 kDa (estimates from the migration in agarose gels at 2 h), TMEM2^ECD^ is estimated to cut at 20 times more sites along the HA during this time period. Thus, the enzymatic activity of TMEM2 appears significantly more potent than that of HYAL2, an observation that seems to be in line with the rapid and pronounced accumulation of HA in *Tmem2* knockout than observed in *Hyal2* knockout (12, 18). These results indicate that the HA-degrading activity of TMEM2 is sufficiently robust for TMEM2 to be considered as a functional hyaluronidase.

### TMEM2 is capable of degrading unlabeled, native HA

As a second additional means of establishing the physiological relevance of TMEM2, we examined the ability of TMEM2^ECD^ to degrade unlabeled, high-molecular weight HA. This determination is necessary, because our results with FA–HAs from commercial sources other than Cosmo Bio have raised a note of caution regarding the use of fluorescence-labeled HA as a surrogate substrate for hyaluronidase assays (see the next section below for these data). Since agarose gel electrophoresis is not suitable for quantitative analysis of unlabeled HA, we instead used size-exclusion chromatography combined with the quantification of HA using the HA-binding protein assay (12). In addition, to enable reliable quantification of HA by this method, the degradation reaction was performed with 20-fold more HA than in the assays with FA–HA. This analysis demonstrates that unlabeled HA (1500–1750 kDa) was degraded by TMEM2^ECD^ into 5 to 10 kDa fragment in a time-dependent manner (Fig. 3*B*). The longer time needed for complete degradation than that seen in assays with FA–HA most likely reflects the lower enzyme/substrate ratio used in this experiment. This result confirms the functional relevance of TMEM2 as an enzyme that degrades native high-molecular weight HA.

### Factors that may confound results obtained in TMEM2 hyaluronidase assays

**Figure 4:**
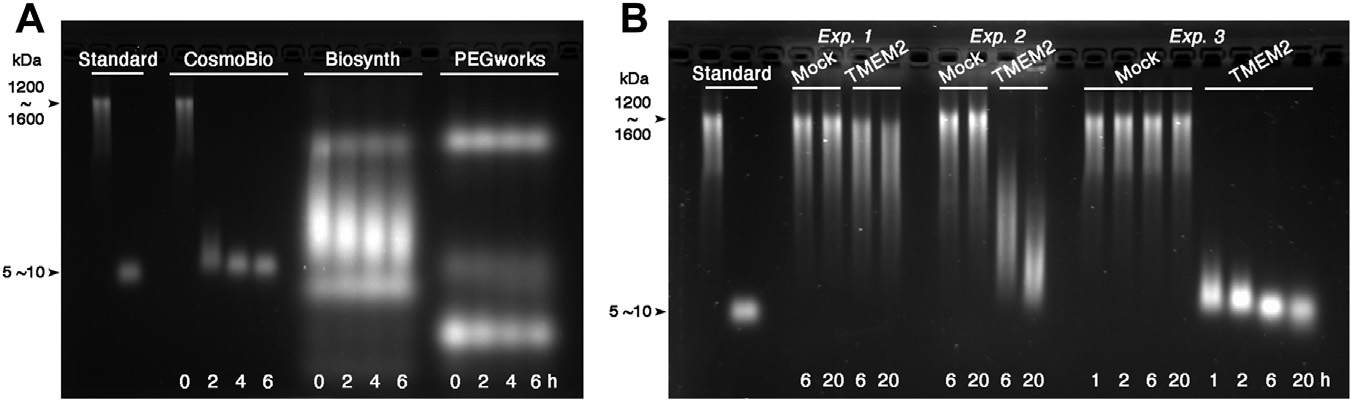
**Identification of factors that confound the TMEM2 hyaluronidase assays.***A*, analysis of the labeled molecular species that comprise the FA–HA products. FA–HAs from Cosmo Bio, Biosynth, and Creative PEGWorks (see Table S1) were analyzed without enzyme treatment or treated with TMEM2^ECD^ for 2, 4, or 6 h. Note that FA–HAs from Biosynth and Creative PEGWorks contain little high-molecular weight HA; instead, they are comprised of multiple species of polydisperse bands (lanes marked *0 h*). These bands are largely unaffected by treatment with TMEM2^ECD^ (lanes marked *2*, *4*, *6 h*). *B*, inhibition of the HA-degrading activity of human TMEM2 by unknown components in 293T cell culture supernatants. HA degradation assays were performed with FA–HA from Cosmo Bio as a substrate using the following samples as enzyme sources: unconcentrated culture supernatants from 293T cells expressing human TMEM2^ECD^ (*Exp. 1*); the same culture supernatants concentrated 30-fold with a 50 kDa cutoff spin column (*Exp. 2*); and 30-fold dilution of the concentrated supernatant used in Exp. 2 with the regular reaction buffer (*Exp. 3*). FA–HA, fluorescein-labeled HA; HA, hyaluronan; TMEM2, transmembrane protein 2; TMEM2^ECD^, ectodomain of TMEM2.

Recently, two papers reported that they could not detect HA-degrading activity in TMEM2 (19, 20). Niu *et al.* (19) reported that recombinant soluble human TMEM2, similar to our TMEM2^ECD^, did not degrade FA–HA. While they employed an assay system similar to ours, a potentially key difference between their experiments and ours is the source of FA–HA products used as a substrate. We have consistently been using FA–HA from Cosmo Bio (11, 12), while Niu *et al.* used FA–HA from Biosynth. Since these FA–HA products differ significantly in both HA size and the degree of substitution with fluorescein moieties (Table S1), we compared these FA–HA products side-by-side, along with a third FA–HA product from Creative PEGWorks. Surprisingly, agarose gel electrophoresis revealed that these three FA–HA products differ markedly in their quality (Fig. 4*A*). While the FA–HA from Cosmo Bio consists of a single high-molecular weight band that is completely degraded by TMEM2^ECD^, the other two FA–HA products contain no components that represent high-molecular weight HA. Instead, they contain multiple species of polydisperse smears and bands that are not affected by TMEM2^ECD^ (Fig. 4*A*) or HYAL2 (Fig. S2). We have thus far been unable to determine the molecular identity of these smears/bands. Since neither TMEM2^ECD^ nor HYAL2 degrades these unidentified smears/bands, they may represent non-HA polysaccharides that contaminate the supposedly “pure” HA used for fluorescein labeling. Another possibility is that differences in fluorescein labeling condition and in the degree of substitution may have generated denatured or partially degraded HA species that are resistant to treatment with these hyaluronidases. In any event, these results emphasize the needs to re-scrutinize earlier data generated by use of these questionable FA–HA products.

In a second contradictory paper, Sato *et al.* (20) reported that they could not detect HA-degrading activity of human TMEM2 using a cell-based assay with transfected 293T cells, although mouse TMEM2 exhibited HA-degrading activity in the same assay. These authors claim that human TMEM2 (in contrast to mouse TMEM2) is not a hyaluronidase. Since we noted earlier in the Results that cell-based HA-degrading assays might contain a factor(s) that suppresses the activity of TMEM2, we analyzed the HA-degrading activity of culture supernatants from human TMEM2^ECD^-transfected 293T cells. While little HA-degrading activity is detected in TMEM2^ECD^-containing culture supernatant, a noticeable HA-degrading activity emerges upon 30-fold concentration of the same culture supernatant using a 50 kDa cutoff spin column (Fig. 4*B*). Remarkably, 30-fold dilution of this concentrated supernatant with the regular reaction buffer results in the emergence of a highly robust activity. These results indicate that although strongly suppressed under the conditions used for cell-based HA-degrading assays, human TMEM2 does indeed possess HA-degrading activity. The suppression of TMEM2 activity must be mediated by an unidentified substance(s) that passes through a 50 kDa cutoff membrane and is then reduced to insignificant levels upon 30-fold dilution. At present, the identity and the origin of the substance(s) are unknown (see Discussion).

## Discussion

Since our first report identifying TMEM2 as a novel cell surface hyaluronidase (11), we have presented extensive data illustrating the functional significance of TMEM2 in systemic HA catabolism (12), cell adhesion and migration (14), and embryonic development (13). Yet, a lack of data directly demonstrating the catalytic activity of purified TMEM2 protein has left some uncertainty about the role TMEM2 as a hyaluronidase. This has led to speculation that TMEM2 may not itself be a hyaluronidase but rather may act as an accessory or regulatory molecule for a known or unknown HA degradation system (20). Here, we present data that convincingly demonstrate the catalytic activity of TMEM2. The key findings of this paper include (i) purified TMEM2^ECD^ exhibits HA-degrading activity without requiring the participation of any other proteins; (ii) this activity is demonstrated in multiple recombinant TMEM2 proteins and assays; (iii) the activity of TMEM2^ECD^ is more potent than that of HYAL2; and (iv) TMEM2^ECD^ degrades native high-molecular weight HA. It should also be stressed that contrary to the recent claim that human TMEM2 lacks HA-degrading catalytic activity (20), our results clearly demonstrate that while somewhat less active than mouse TMEM2, human TMEM2 possesses intrinsic HA-degrading activity that is more potent than that of human HYAL2.

In addition to these core findings, our study has revealed factors that may confound the results of TMEM2 hyaluronidase assays. One such factor is the use of FA–HA as a substrate. As revealed in this study, the quality of FA–HA is vastly different among products from different vendors. From the migration patterns in agarose gel, the FA–HA products from sources other than Cosmo Bio consist of multiple fluorescein-labeled bands and smears that do not appear to be intact HA. Those poorly characterized molecular species may represent degraded or denatured HA or even molecules totally unrelated to HA. Since the vendors of FA–HA do not provide detailed information regarding the source and purification of HA species used for labeling, it is difficult to speculate about the cause(s) for these differences in FA–HA quality. However, it is known that bacteria used for large-scale fermentation for HA production, such as *Streptococci*, can produce not only HA but also other nonsulfated glycosaminoglycans (21, 22). Also, a high degree of substitution with fluorescein labels, as seen in the FA–HA from Biosynth, could affect the recognizability and/or cleavability by TMEM2, as the fluorescent labels can significantly affect the chemical properties of labeled molecules (23, 24). Although we cannot rule out the possibility that the problematic nature of the Biosynth FA–HA is specific to the lot we obtained, our results nonetheless emphasize the importance of a careful quality check of purchased FA–HA products, as well as the need to re-examine earlier data generated *via* use of these FA–HA products.

Another potentially confounding factor uncovered in this study is the inhibitory effect of unknown components present in cell-based HA degradation assays. This is most likely the reason why our earlier experiments required a much longer time course for complete HA degradation (11) than we observe in assays with purified TMEM2^ECD^. The failure of Sato *et al.* (20) to detect the catalytic activity of human TMEM2 might also have been due to this issue. The identity of the inhibitory substance(s) and its origin, whether it is a component(s) of culture media or a cell-derived factor, is currently unknown. Standard components of cell culture media, such as vitamins and sodium pyruvate, have been shown to inhibit the activity of various enzymes (25, 26, 27, 28, 29, 30, 31, 32, 33, 34). Inorganic anions in culture media, serum added to media, and lactate produced by cells could all reduce the effective concentration of calcium (35), a necessary factor for TMEM2 activity (11). The possibility that 293T cells secrete a biological TMEM2 inhibitor(s) cannot be ruled out at present.

Lastly, although not specifically investigated in this study, it should be noted that, when FA–HA is used as a substrate for hyaluronidase assays, agarose gel electrophoresis is preferable to size-exclusion chromatography for the analysis of the degradation products. Agarose gel electrophoresis allows the analysis of HA fragments over the entire molecular weight range that needs to be covered. In contrast, size-exclusion chromatography using a resin with a small pore size, such as Sephacryl S-200, poses a risk of missing partial HA degradation in the high-molecular weight range.

In conclusion, our results clearly demonstrate that TMEM2 is a *bona fide* hyaluronidase that possesses an intrinsic and physiologically relevant activity. While the mechanistic details of TMEM2-mediated HA degradation on the cell surface remain to be determined, these new findings are important in resolving uncertainties that have surrounded the role of TMEM2 as a hyaluronidase. This resolution should facilitate research on this intriguing molecule that is thought to play important roles in embryonic development (13, 36, 37, 38), tumor progression (39, 40), and aging (41).

## Experimental procedures

### Materials

For this study, three FA–HA products from different manufacturers were used as substrates for HA degradation assays, namely: CSR-FAHA-H2 from Cosmo Bio; YH4531 from Biosynth; and HA-804 from Creative PEGWorks. Unlabeled HA (1500 ∼ 1750 kDa) was obtained from Sigma (63357), while FA–HA molecular weight standards (CSR-FAHA-H2, CSR-FAHA-L2, and CSR-FAHA-U2) were from Cosmo Bio. Recombinant soluble human HYAL2 protein was purchased from R&D Systems (11012-GH-020).

### Production of recombinant TMEM2^ECD^ proteins

Expression plasmid for human TMEM2^ECD^ fused with a C-terminal 6× His tag was constructed by inserting the cDNA into pSecTag2B, in which the original Myc/His tag was inactivated by inserting a stop codon. For the production of mouse TMEM2^ECD^, the construct used for the expression of a tag-less TMEM2 ectodomain (11) was modified by inserting a 6× His tag at its C terminus. TMEM2^ECD^ proteins were produced using the FreeStyle 293-F system (ThermoFisher, R79007), as described previously (42). Culture supernatants were first applied to HisPur Cobalt Resin (ThermoFisher, R79007), and bound TMEM2^ECD^ proteins were eluted with PBS containing 0.5 M imidazole and 10% glycerol. TMEM2^ECD^ proteins were further purified by size-exclusion chromatography on HiLoad 26/600 Superdex 200 (Cytiva, 28989336). Concentration of purified TMEM2^ECD^ proteins was determined by measuring absorbance at 280 nm.

### Hyaluronidase assays

For assays with FA–HA as substrates, 2.5 μg of purified TMEM2^ECD^ was incubated at 37 °C with 0.25 μg FA–HA in 500 μl of 50 mM Hepes buffer (pH 7.0) containing 2 mM CaCl_2_ (“reaction buffer”) at 37 °C. For assays with unlabeled HA as substrates, 10 μg of purified human TMEM2^ECD^ was incubated with 5 μg HA in the reaction buffer at 37 °C. In the comparative analysis of N- and C-terminally 6× His-tagged TMEM2^ECD^ proteins and the analysis of TMEM2 inhibition, 293T cells were transfected with respective TMEM2^ECD^ constructs and cultured in Opti-MEM serum-free medium for 3 days. After a 30-fold concentration using Vivaspin 6 (50 kDa cutoff, Cytiva 28932294), the concentrated culture supernatants were used as enzyme sources in HA degradation assays. In the case of the former analysis, the input of N- and C-terminally tagged TMEM2^ECD^ proteins was normalized based on the densitometric analysis of immunoblots with anti-TMEM2 polyclonal antibody (Invitrogen PA5-85901) using ChemiDoc MP Imaging System (Bio-Rad). For assays with full-length TMEM2 in the membrane fraction, C-terminally FLAG-tagged TMEM2 were transfected into 293T cells, from which the P2 membrane fraction was prepared as described (11) and used as enzyme source. In all these experiments, the enzyme reaction was terminated by adding formamide (final 10 M). In experiments using FA–HAs as substrate, degradation of HA was analyzed by agarose gel electrophoresis in 1% SeaKem GTG (Lonza 50070) or size-exclusion chromatography on Sephacryl S-300HR (Cytiva 17059910) (11, 12). Degradation of unlabeled HA was analyzed by size-exclusion chromatography on Sepharose CL-2B (Cytiva 17014001), combined with the quantification of HA using the Hyaluronic Acid LT Assay (Fujifilm Wako Chemicals). The ability of TMEM2-transfected cells to degrade substrate-bound HA was analyzed by *in situ* HA degradation assays, as described (11, 14).

## Data availability

All data are contained within the article and supporting information.

## Supporting information

This article contains supporting information.

## Conflict of interest

The authors declare that they have no conflicts of interest with the contents of this article.
