# Supplementary material for: TMEM2 is a bona fide hyaluronidase possessing intrinsic catalytic activity
Source: J Biol Chem. 2023 Jul 30;299(9):105120. doi: 10.1016/j.jbc.2023.105120 (PMC10474455; doi:10.1016/j.jbc.2023.105120)
Supplement: Table S1 [file mmc1.pdf]

Table S1. FA-HAs used in this study.

Vendor	Catalog #	Lot #	Size (kDa)	Labeling site	Degree of substitution (mol %)
**Cosmo Bio**	CSR-FAHA-H2	21K702	1200~1600	-COOH (GlcU*)	0.6~1.1
**Biosynth**	YH45321	453211501	800	-COOH (GlcU*)	4.0~6.0
**Creative PEGWorks**	HA-804	WX80403312015	1500	-COOH (GlcU*)	1.0

*GlcU: glucuronic acid
