# Supplementary material for: TMEM2 is a bona fide hyaluronidase possessing intrinsic catalytic activity
Source: J Biol Chem. 2023 Jul 30;299(9):105120. doi: 10.1016/j.jbc.2023.105120 (PMC10474455; doi:10.1016/j.jbc.2023.105120)
Supplement: Figures S1 and S2 [file mmc2.pdf]

Ig signal sequence | 6x His | Ser104 ~ His1383

N-His: METDTLLLWVLLLWVPGSTGDAAQHHHHHHTKLGTSSKYAPDE.........LKQASKAH

Linker (AAQ) | Linker (TKLGT)

Ig signal sequence | Ser104 ~ His1383 | 6x His

C-His: METDTLLLWVLLLWVPGSTGDAAQPARRARRTKLGTSSKYAPDE.........LKQASKAHGSSENLYFQGSSHHHHHH

Linker (AAQPARRARRTKLGT) | Linker (GSSENLYFQGSS)

Figure S1. Amino acid sequence of recombinant human TMEM2^{ECD} proteins. *N-His*, N-terminally 6x His-tagged TMEM2^{ECD}; *C-His*, C-terminally 6x His-tagged TMEM2^{ECD}.

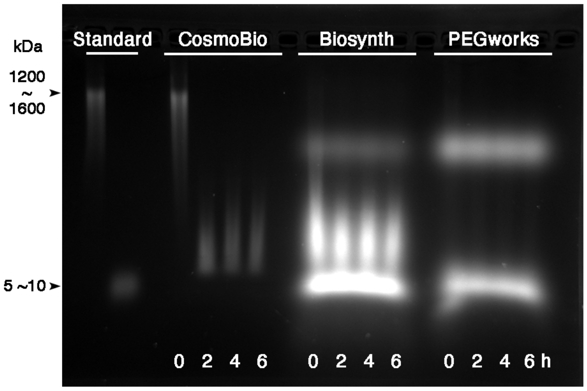

Figure S2. Effects of recombinant human HYAL2 on FA-HAs from three different vendors. Experiments were performed by the same procedure as described in Fig. 4A. Note that the FA-HAs from Biosynth and Creative PEGWorks are comprised of multiple species of polydisperse bands (lanes marked *0 h*), and that these unidentified bands are not degraded by HYAL2 (lanes marked *2, 4, 6 h*).
